# Electroacupuncture versus 5-HT4 receptor agonist for functional constipation: A systematic review and meta-analysis of randomized controlled trials

**DOI:** 10.1097/MD.0000000000040634

**Published:** 2024-11-29

**Authors:** Shanchun Xu, Jiacheng Li, Aimei Wang

**Affiliations:** a Department of General Medicine, The First Affiliated Hospital of Zhejiang Chinese Medical University (Zhejiang Provincial Hospital of Chinese Medicine), Hangzhou, China; b Department of Colorectal Surgery, Suzhou TCM Hospital Affiliated to Nanjing University of Chinese Medicine, Suzhou, China; c Department of Oncology, The Affiliated Taizhou People’s Hospital of Nanjing Medical University, Taizhou, China.

**Keywords:** complementary medicine, constipation, electroacupuncture, meta-analysis, review

## Abstract

**Background:**

Functional constipation (FC) has been found as a chronic gastrointestinal disease that is commonly diagnosed in patients. However, patients have a low satisfaction level with the treatment of constipation drugs (e.g., 5-HT4 agonists). A meta-analysis was performed to compare the efficacy and safety between electroacupuncture and 5-HT4 agonists.

**Methods:**

The included study were randomized controlled trials (RCTs), in which EA was used in the experimental group and 5-HT4 receptor agonist was used in the control group. Four English databases (PubMed, Cochrane Library, Web of Science, Embase) and 4 Chinese databases (China National Knowledge Infrastructure, CBM, WanFang, VIP) were searched. Relevant studies retrieved were published before September 30, 2024. The risk of bias was assessed by tool of Cochrane and GRADEpro. The Review Manager 5.4 was used for analyzing Data analysis, and Endnote X9 for screening studies.

**Results:**

In this paper, we included 12 studies, involving 1473 participants. We found that EA significantly improved patient assessment of cab quality of life questionnaire (PAC-QOL) (MD = −0.52, *P* = .03), self-rating anxiety scale (SAS) (MD = −3.00, *P* < .00001) and self-rating depression scale (SDS) (MD = −4.13, *P* < .00001) compared with 5-HT4 receptor agonists. In addition, we failed to identify any significant difference in Stool consistency, the number of weekly complete spontaneous bowel movements and weekly spontaneous bowel movements (SBMs) between the 2 groups.

**Conclusion:**

EA has been indicated to be better than 5-HT4 receptor agonists since it can more effectively improve FC patients’ life quality and mental state without an increased risk of adverse even. However, the previous evidence is characterized by low quality and small sample size, which should be further confirmed by high-quality and large-sample multicenter RCTs.

## 1. Introduction

Functional constipation (FC) is a highly prevalent functional gastrointestinal disorder among adults. FC is characterized by difficult and infrequent bowel movements.^[1]^ According to a meta-analysis, the global prevalence of FC is 14%, with higher rates observed in women and the elderly.^[2]^ However, the prevalence of FC is 7.8% in Western countries, 8.76% in Japan.^[3,4]^ Importantly, the prevalence is 8.2% in China.^[5]^ Patients suffering from FC are accompanied by anxiety with poor quality of life (QOL).^[6,7]^ This gastrointestinal disorder adversely affects both the physical and emotional health of patients.

Thus far, the treatment of FC mainly covers pharmacological treatments and non-pharmacological treatments. 5-HT4 receptor agonists, 1 of the drugs for the treatment of constipation, have been extensively used clinically,^[8]^ which can facilitate the release of acetylcholine from cholinergic neurons in the intestine to achieve the purpose of stimulating the gut motility.^[9]^ Among them, the evidence-based medicine evidence of prucalopride is Level I, grade A.^[10]^ However, it may occasionally have some side effects during treatments, including bloating,^[11]^ for better clinical efficacy and fewer adverse effects, nondrug therapies including fecal bacteria transplantation,^[12]^ biofeedback,^[13]^ baduanjin,^[14]^ moxibustion,^[15]^ and acupuncture^[16]^ become novel options for FC patients.

Acupuncture refers to an alternative medicine from the East. The latest study reported that Electroacupuncture stimulation drive the vagal-adrenal anti-inflammatory axis in mice. Acupuncture lays a modern neuroanatomical basis for the relatively specific existence of acupuncture points.^[17]^ Impacted by the publication of this research, electroacupuncture has once again attracted wide attention. The process of electroacupuncture treatment involves 3 steps: inserting the needle into the corresponding acupuncture point by hand, performing small and equal rotation, lifting, and thrusting operations on the needle to achieve Qi (a compound sensation that includes pain, numbness, swelling, heaviness, and other sensations), and connecting the electrode to the needle handle, turning on the current, and continuing the stimulation for 30 minutes.^[18]^ Electroacupuncture increases completely spontaneous bowel movements, which has been demonstrated by RCT.^[19]^ Thus far, EA mechanism on FC is still unclear, whereas animal experiments reported that EA up-regulates TPH expression within colon tissues.^[20]^ Some reported that EA is likely to increase SP mRNA expression in the hypothalamus and distal colon and improve the intestinal microflora structure.^[21]^ EA may become 1 of the better options for FC patients.

The choice between electroacupuncture (EA) and 5-HT4 receptor agonists for treating FC remains controversial. Few systematic reviews have addressed randomized controlled trials (RCTs) comparing EA with 5-HT4 receptor agonists for FC treatment. Given the long history of acupuncture in treating FC and the substantial amount of RCT evidence, this meta-analysis aims to critically assess the efficacy and safety of EA versus 5-HT4 receptor agonists in treating FC patients.

## 2. Methods

### 2.1. Date sources and search criteria

RCTs were comprehensively sourced from 4 English databases (PubMed, Cochrane Library, Web of Science, Embase) and 4 Chinese databases [Wanfang Data, Chinese Biomedical Literature Database (CBM), Chinese Science Journal Database (VIP), China National Knowledge Infrastructure]. Literature published before September 30, 2024, was independently searched by 2 reviewers. This paper integrated the keywords according to MeSH headings with self-generated keywords for study screening, based on the limitations of Chinese or English language.

### 2.2. Search strategy

#### 2.2.1. Patient

#1 MeSH descriptor: [Constipation] explode all trees.

#2 “FC”: ti,ab,kw OR “Dyschezia”:ti,ab,kw OR “Colonic Inertiar”:ti,ab,kw OR “chronic constipation”:ti,ab,kw.

#3 #1 OR #2.

#### 2.2.2. Interventions

#4 MeSH descriptor: [Electroacupuncture] explode all trees.

#5 “electroacupuncture”: ti,ab,kw OR “electro-acupuncture”:ti,ab,kw.

#6 #4 OR #5.

#### 2.2.3. Study design

#7 MeSH descriptor: [randomized controlled trial] explode all trees.

#8 “Randomized controlled trial”: ti,ab,kw OR “controlled clinical trial”:ti,ab,kw OR random*:ti,ab,kw OR placebo*:ti,ab,kw OR “RCT”:ti,ab,kw.

#9 #7 OR #8.

#10 #3 AND #6 AND #9 (Mesh terms in Table 1).

**Table 1:** Search strategy.

Database source	Patient	Interventions	Study design
The Cochrane library	#1MeSH descriptor: [Constipation] explode all trees #2“functional constipation”:ti,ab,kw OR “Dyschezia”:ti,ab,kw OR “Colonic Inertiar”:ti,ab,kw OR “chronic constipation”:ti,ab,kw #3 #1 OR #2	#4MeSH descriptor: [Electroacupuncture] explode all trees #5“electroacupuncture”: ti,ab,kw OR “electroacupuncture”:ti,ab,kw #6 #4 OR #5	#7MeSH descriptor: [Randomized Controlled Trial] explode all trees #8“Randomized controlled trial”: ti,ab,kw OR “controlled clinical trial”:ti,ab,kw OR random*:ti,ab,kw OR placebo*:ti,ab,kw OR “RCT”:ti,ab,kw #9 #7 OR #8 #10 #3 AND #6 AND #9
*PubMed*	#1 “Constipation” [Mesh] #2 Colonic Inertia [Title/Abstract] OR “functional constipation” OR “Dyschezia”[Title/Abstract] OR “chronic constipation”[Title/Abstract] #3 #1 OR #2	#4“Electroacupuncture” [Mesh] #5 “Electro-acupuncture” [Title/Abstract] #6 #4 OR #5	#7’randomized controlled trial (topic)’/exp #8′randomized controlled trial’:ab,ti OR “controlled clinical trial”:ab,ti OR “placebo”:ab,ti OR random*:ab,ti OR RCT:ab,ti #9 #7 OR #8 #10 #3 AND #6 AND #9
*EMBASE*	#1 “constipation”/exp #2 functional constipation:ab,ti OR “chronic constipation”:ab,ti OR “Dyschezia”:ab,ti OR “Colonic Inertia”:ab,ti #3 #1 OR #2	#4’electroacupuncture’/exp #5’electroacupuncture’: ab,ti #6 #4 OR #5	#7’randomized controlled trial (topic)’/exp #8′randomized controlled trial’:ab,ti OR “controlled clinical trial”:ab,ti OR “placebo”:ab,ti OR random*:ab,ti OR RCT:ab,ti #9 #7 OR #8 #10 #3 AND #6 AND #9
*Web of Science*	#1 constipation OR functional constipation OR chronic constipation OR Colonic Inertia OR Dyschezia	#2Electroacupuncture OR electroacupuncture	#3randomized controlled trial OR controlled clinical trial OR random* OR placebo OR RCT #4 #1 AND #2 AND #3
*Chinese Database (CNKI, CBM, WanFang, VIP*)	#1 “便秘” OR “慢性便秘” OR “慢性功能性便秘” OR “功能性便秘” OR “慢性严重便秘”	#2 “电针” OR “电针疗法”	#3 “随机对照试验” OR “随机对照试验” OR “随机对照研究” OR “RCT” OR “随机对照” OR “随机” #4 #1 AND #2 AND #3

### 2.3. Inclusion and exclusion criteria

We have developed inclusion and exclusion standards. The inclusion standards below were employed here: The subjects were FC patients satisfying Rome III diagnostic criteria.^[22]^ The treatment group received the treatment using electroacupuncture, and the control received the treatment using 5-HT4 receptor agonists (e.g., mosapride and prucalopride). We did not restrict the acupoints, dosage, treatment period, frequency, course of treatment, and route of administration of electroacupuncture. The research design is a RCT. The studies below were excluded: studies concerned with drug-related constipation patients or patients with constipation secondary to other diseases; patients with electroacupuncture combined with other therapies intervention; animal experiments, case reports, reviews, programs, and comments research with unreported data; Repeated research with the same content.

### 2.4. Outcome measures

The primary outcome was the numbers of spontaneous bowel movement (SBM) as well as complete spontaneous bowel movement (CSBM). The former represents the number of times of spontaneous and complete defecation without relying on laxatives or manipulations, and the latter represents the number of stools without relying on cessation. The number of bowel movements and other laxative methods was determined according to the number of times of going to the toilet The secondary outcomes were stool consistency, at the Bristol stool form scale^[23]^; patient assessment of constipation quality of life questionnaire (PAC-QOL)^[24]^; self-rating anxiety scale (SAS) and self-rating depression scale (SDS)^[25]^; adverse events of EA and 5-HT4 receptor agonist for treating constipation.

### 2.5. Study selection and data extraction

The screening of RCT was made by 2 independent reviewers in accordance with the predetermined criteria. The study characteristics and exclusion reasons were recorded using the Excel datasheet. The disagreement was resolved based on a discussion or by consulting a third reviewer. Data extraction was carried out for the final included studies. Basic information consisted of general information (e.g., sample size, publication date and first author), patient features (e.g., gender, age, duration of treatment and duration of follow-up), intervention method, outcome measures (e.g., complete spontaneous bowel movements (CSBMs), SBMs, stool consistency, PAC-QOL, SAS, and SDS), and adverse events.

### 2.6. Quality assessment

In accordance with the Cochrane risk of bias tool, 2 independent reviewers carried out an assessment of all included RCTs. The risk of bias was assessed according to 7 criteria: selective reporting (reporting bias), incomplete outcome data (attrition bias), blinding of outcome assessment (detection bias), blinding of participants and personnel (performance bias), allocation concealment (selection bias), random sequence generation (selection bias), and other potential sources of bias. Each domain was categorized as unclear, high, or low risk. Any disagreements were resolved through discussion or by consulting a third reviewer.

### 2.7. Data synthesis and statistical investigation

Software Review Manager (Revman 5.4) developed by Cochrane Collaborations was used for data analysis. Random effects model was used for each outcome comparison. The results were expressed to be a 95% confidence interval (CI) for binary data and a weighted mean difference for continuous information, or a 95% CI for mean difference (MD). If *P* < .05, the difference of the experimental group and the control was statistically significant.

### 2.8. Grading of evidence quality

This paper employed GRADEpro (Grading of Recommendations Assessment, Development and Evaluation Working Group) software to classify the evidence quality, which falls into very low, medium, low and high evidence quality. Five downgrading factors should be considered (i.e., the imprecision, indirectness, inconsistency, risk of bias, and other biases in the study).

## 3. Results

### 3.1. Study selection

**Figure 1.**
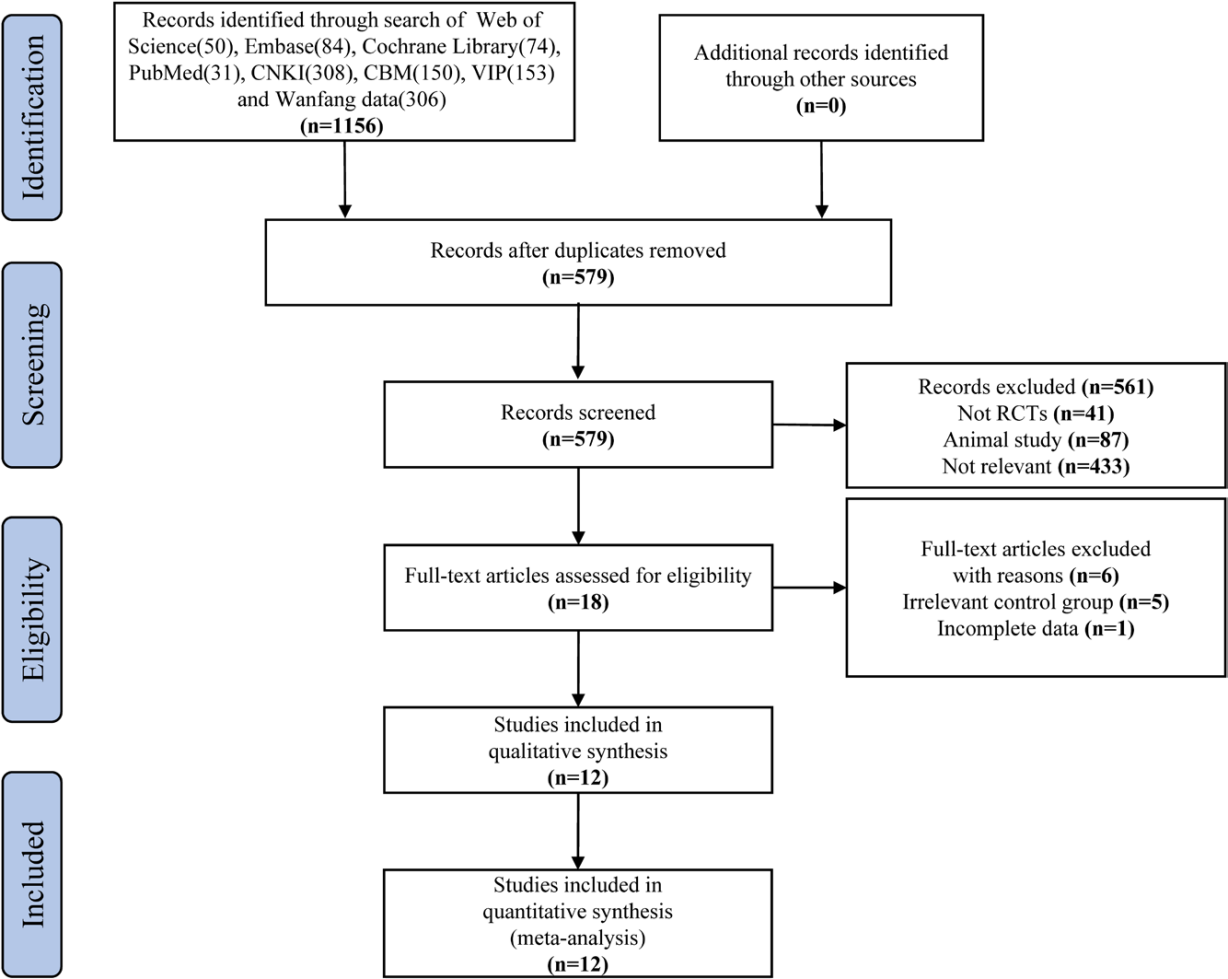
Selection process of the study.

In accordance with the search strategy formulated above, 1156 studies were retrieved in total from the 8 databases (Fig. 1). After deleting duplicates, we screened out 579 studies by consensus. To be specific, we excluded 561 studies after a preliminary screening by title and abstract. Subsequently, we selected the finalists based on the full text obtained. 6 studies interventions were problematic (e.g., inconsistent EA), and 1 data was incomplete. Lastly, 12 eligible RCTs^[26–37]^ with 1473 subjects in total were recruited for the following meta-analysis.

### 3.2. Basic characteristics of included trials

The 12 included studies were all conducted in China, of which 8 were published in Chinese and 4 were published in English. All studies compared EA in the intervention group with 5-HT4 receptor agonists in the control. Moreover, all studies included found no significant difference at baseline, and the studies were published between 2013 and 2021. The duration of treatment lasted from 4 to 8 weeks, with 4 weeks as the main one (8/12, 66.67%). Table 2 lists the basic features of 12 included researches.

**Table 2:** Basic characteristics of the included 12 RCTs.

Study	Sample size (T/C)	Sex (M/F)	Age (yr)	Constipation duration (yr)	Intervention	Control	Treatment duration (wk)	Follow-up period (wk)	Outcomes
Peng et al (2013)^[28]^	T: 30 C: 30	8/22 10/20	42.36 ± 3.88 41.82 ± 4.06	n.r. n.r.	EA ST25, ST36	Mosapride cirate	4	n.r.	SAS, SDS
Xiong et al (2014a)^[29]^	T: 35 C: 38	4/31 3/35	30.29 ± 12.95 30.77 ± 12.41	8.17 ± 7.26 7.74 ± 6.59	LIS of EA LI11, ST37	Mosapride cirate	4	n.r.	SAS, SDS
Xiong et al (2014b)^[29]^	T: 38 C: 38	4/34 3/35	30.49 ± 15.89 30.77 ± 12.41	9.12 ± 9.76 7.74 ± 6.59	HIS of EA LI11, ST37	Mosapride cirate	4	n.r.	SAS, SDS
Wu et al (2014a)^[30]^	T: 19 C: 25	7/12 6/19	n.r. 55 ± 11	n.r. n.r.	EA ST25, BL25	Mosapride cirate	4	4	Weekly SBMs, AEs
Wu et al (2014b)^[30]^	T: 34 C: 25	7/27 6/19	53 ± 12 55 ± 11	n.r. n.r.	EA LI11, ST37	Mosapride cirate	4	4	Weekly SBMs, AEs
Wu et al (2014c)^[30]^	T: 26 C: 25	7/19 6/19	56 ± 9 55 ± 11	n.r. 18.11 ± 10.44	EA LI11, ST37, ST25, BL25	Mosapride cirate	4	4	Weekly SBMs, AEs,
Xiong et al (2014)^[31]^	T: 58 C: 34	n.r. n.r.	19 to 65 19 to 64	7m-20 8m-19	EA LI11, ST37	Mosapride cirate	4	8	Weekly SBMs, SAS, SDS
Mao et al (2016)^[27]^	T: 20 C: 20	6/14 8/12	44.85 ± 7.71 46.95 ± 9.83	3.69 ± 2.42 3.90 ± 2.75	EA ST25, ST37, SP14	Prucalopride	8	8	Weekly CBMs, Weekly SBMs, Stool consistency
Ding et al (2017)^[32]^	T: 33 C: 30	23/40	34.86 ± 11.76	5.71 ± 2.54	EA LI11, ST37, ST25, BL25	Mosapride cirate	4	4	Weekly SBMs, Stool consistency
Kuang et al (2017)^[33]^	T: 19 C: 19	0/20 0/20	41.53 ± 16.15 35.29 ± 13.26	6.39 ± 0.65 6.33 ± 0.41	EA ST25, ST37, SP14	Prucalopride	8	n.r.	PAC-QOL
Wu et al (2017a)^[34]^	T: 58 C: 67	6/52 10/57	35.00 ± 15.62 43.60 ± 17.90	5.87 ± 7.13 5.67 ± 6.18	LCI of EA LI11, ST37	Mosapride cirate	4	4	Weekly SBMs, PAC-QOL, AEs, Stool consistency
Wu et al (2017b)^[34]^	T: 65 C: 67	5/60 10/57	37.20 ± 18.19 43.60 ± 17.90	7.19 ± 8.67 5.67 ± 6.18	HCI of EA LI11, ST37	Mosapride cirate	4	4	Weekly SBMs, PAC-QOL, AEs, Stool consistency
Zhong et al (2018)^[35]^	T: 36 C: 36	27/9 31/5	30.46 ± 12.88 30.19 ± 9.73	5.22 ± 6.76 3.81 ± 2.65	EA LI11, ST37	Mosapride cirate	4	4	Weekly SBMs, SAS SDS, Stool consistency
Xu et al (2020)^[37]^	T: 31 C: 33	6/25 6/27	35.30 ± 19.10 35.40 ± 15.30	8.90 ± 7.20 8.80 ± 7.70	EA LI11, ST37	Mosapride cirate	4	4	Weekly SBMs, AEs, Stool consistency, SAS, SDS, PAC-QOL
Li et al (2020)^[36]^	T: 42 C: 42	0/42 0/42	46.38 ± 3.19 46.26 ± 3.63	n.r. n.r.	EA ST25, ST37, SP14	Prucalopride	8	n.r.	PAC-QOL
Liu et al (2021)^[26]^	T: 277 C: 278	55/222 54/224	46.06 ± 16.12 45.79 ± 16.00	9.18 ± 8.53 9.03 ± 8.77	EA ST25, ST37, SP14	Prucalopride	8	24	Weekly SBMs, AEs, Stool consistency

Abbreviations: AEs = adverse events, BL25 = acupoints of Dachangshu, CBMs = complete spontaneous bowel movements, EA = electroacupuncture; HIS = high intensity stimulation; HCI = high current intensity; LCI = low current intensity; LIS = low intensity stimulation; LI11 = acupoints of Quchi; m = month; n.r.= not reported; PAC-QOL = patient assessment of constipation quality of life; SAS = self-rating anxiety scale; SBMs = spontaneous bowel movements; SDS = self-rating depression scale; SP14 = acupoints of Fujie; ST25 = acupoints of Tianshu, ST36 = acupoints of Zusanli; ST37 = acupoints of Shangjuxu.

### 3.3. Quality assessment

**Figure 2.**
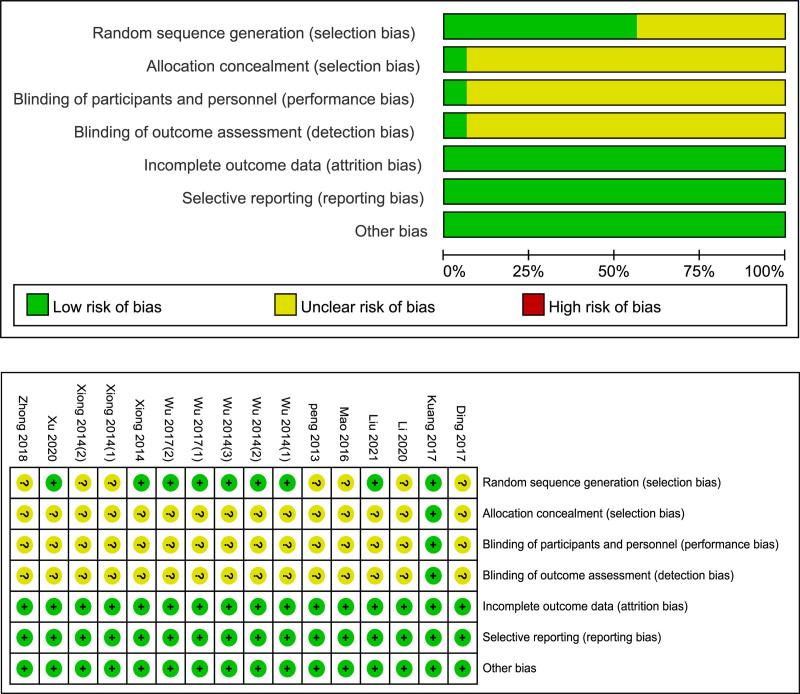
Risk of bias graph and bias summary.

Figure 2 presents the risk of bias graph and summary. In the generation of randomization sequence, we evaluated half of the studies as “low risk” through random number tables or computer-generated random numbers, and the other studies were assessed as “unclear risk” since they did not describe a clear randomization method. One of the studies adopted a triple-blind method. Complete outcome data was found in all studies.

### 3.4. Complete spontaneous bowel movement

**Figure 3.**
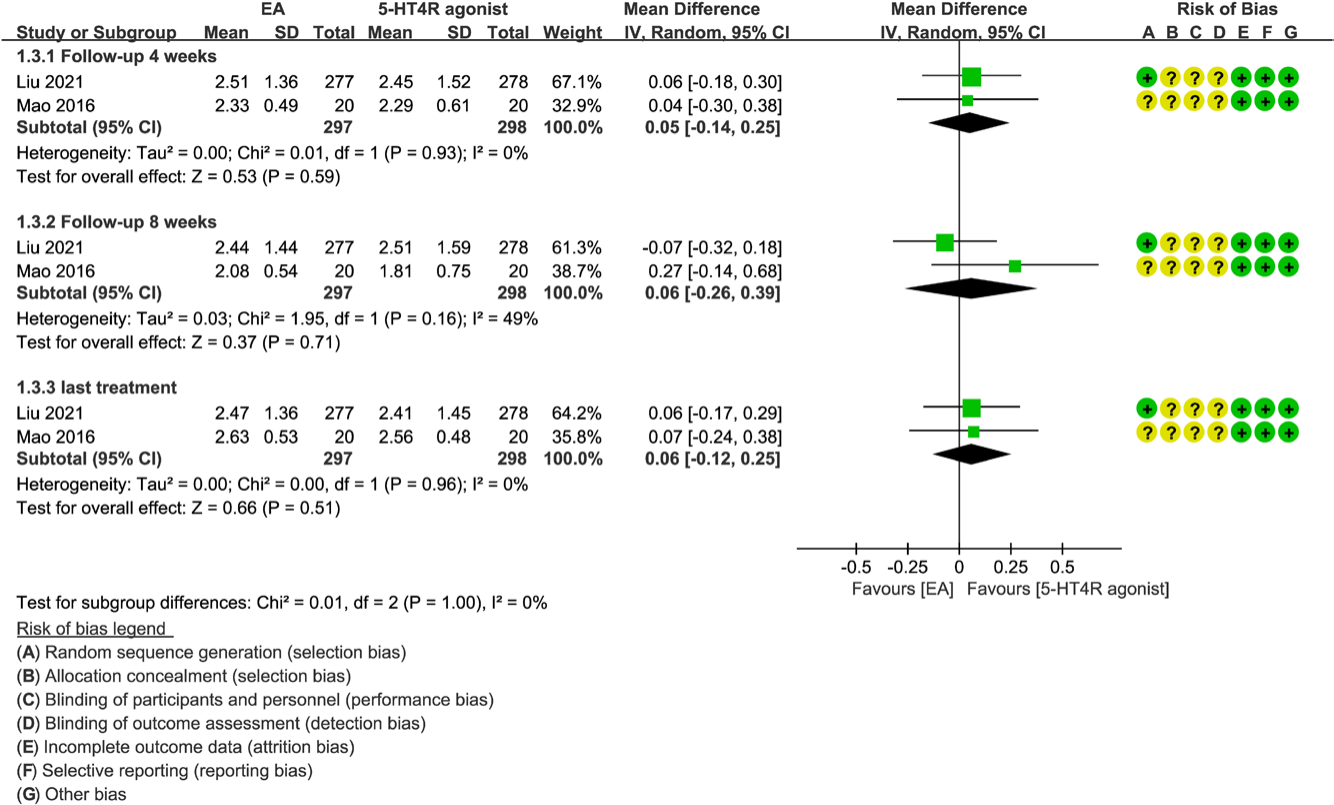
Forest plot of EA versus 5-HT4 receptor agonist weekly CSBMs (follow-up 4 weeks, 8 weeks and last treatment).

Two studies^[26,27]^ (including 595 patients) assessed the improvement of weekly CSBMs in patients in the EA group and the control. In particular, we compared weekly CSBMs at different nodes (treatment and follow-up) between groups. A comparison was drawn for the weekly CSBMs of follow-up 4 weeks, follow-up 8 weeks and the last treatment. In all 3 sets of data, the weekly CSBMs of the EA group were higher, whereas none of them had statistical significance (MD = 0.05; 95% CI = −0.14 to 0.25, *P* = .59; MD = 0.06; 95% CI = −0.26 to 0.39, *P* = .71; MD = 0.06; 95% CI = −0.12 to 0.25, *P* = .51, respectively) (Fig. 3).

### 3.5. Spontaneous bowel movement

**Figure 4.**
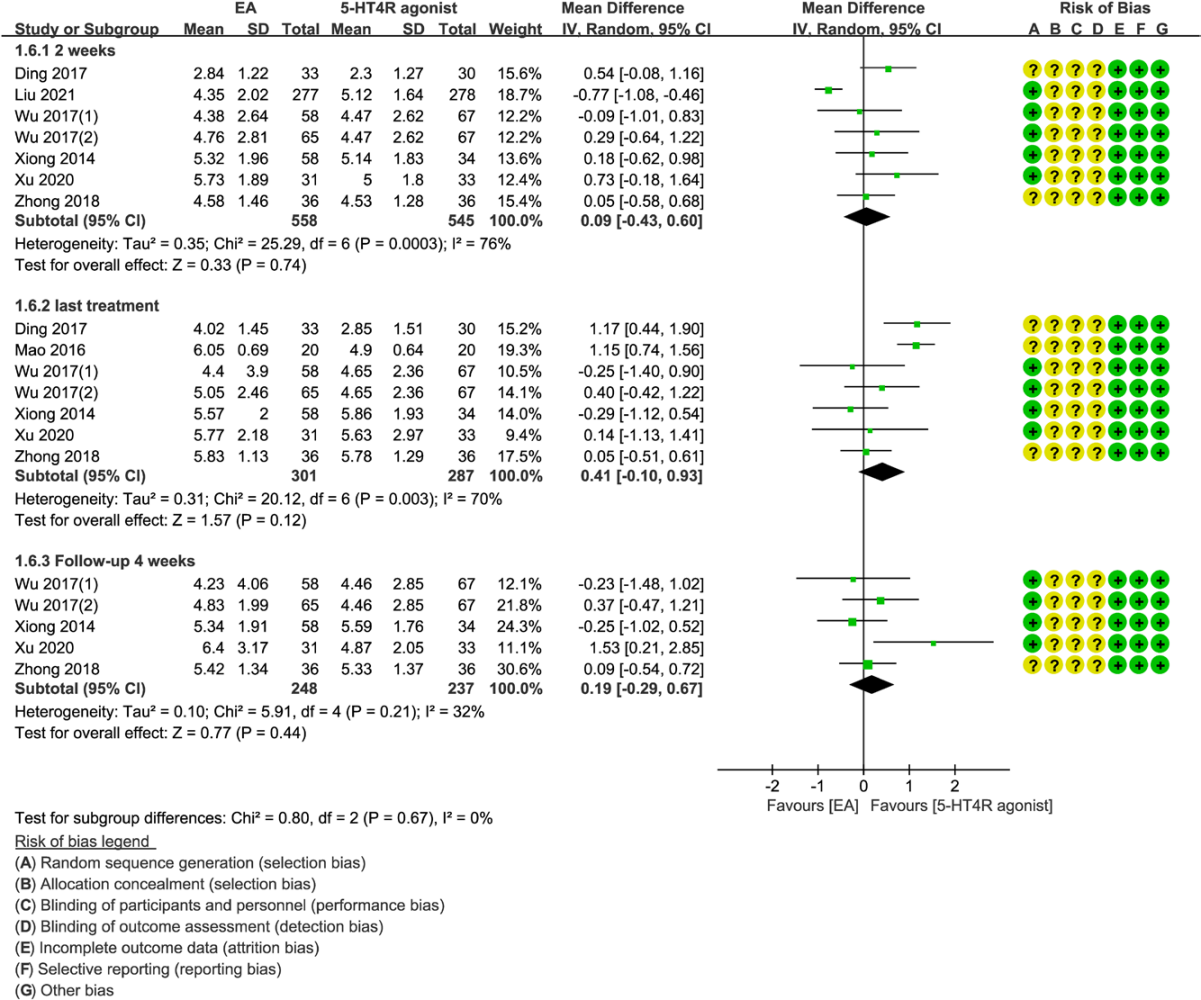
Forest plot of EA versus 5-HT4 receptor agonist weekly SBMs (2 weeks, last treatment and follow-up 4 weeks).

Likewise, we divided the data into 3 groups for analysis according to the duration of treatment and follow-up time. The EA group had more weekly CSBMs than the control. However, the difference between the 3 groups did not have statistical significance (MD = 0.09; 95% CI = −0.43 to 0.60, *P* = .74; MD = 0.41; 95% CI = −0.10 to 0.93, *P* = .12; MD = 0.19; 95% CI = −0.29 to 0.67, *P* = .44, respectively) (Fig. 4).

### 3.6. Stool consistency

**Figure 5.**
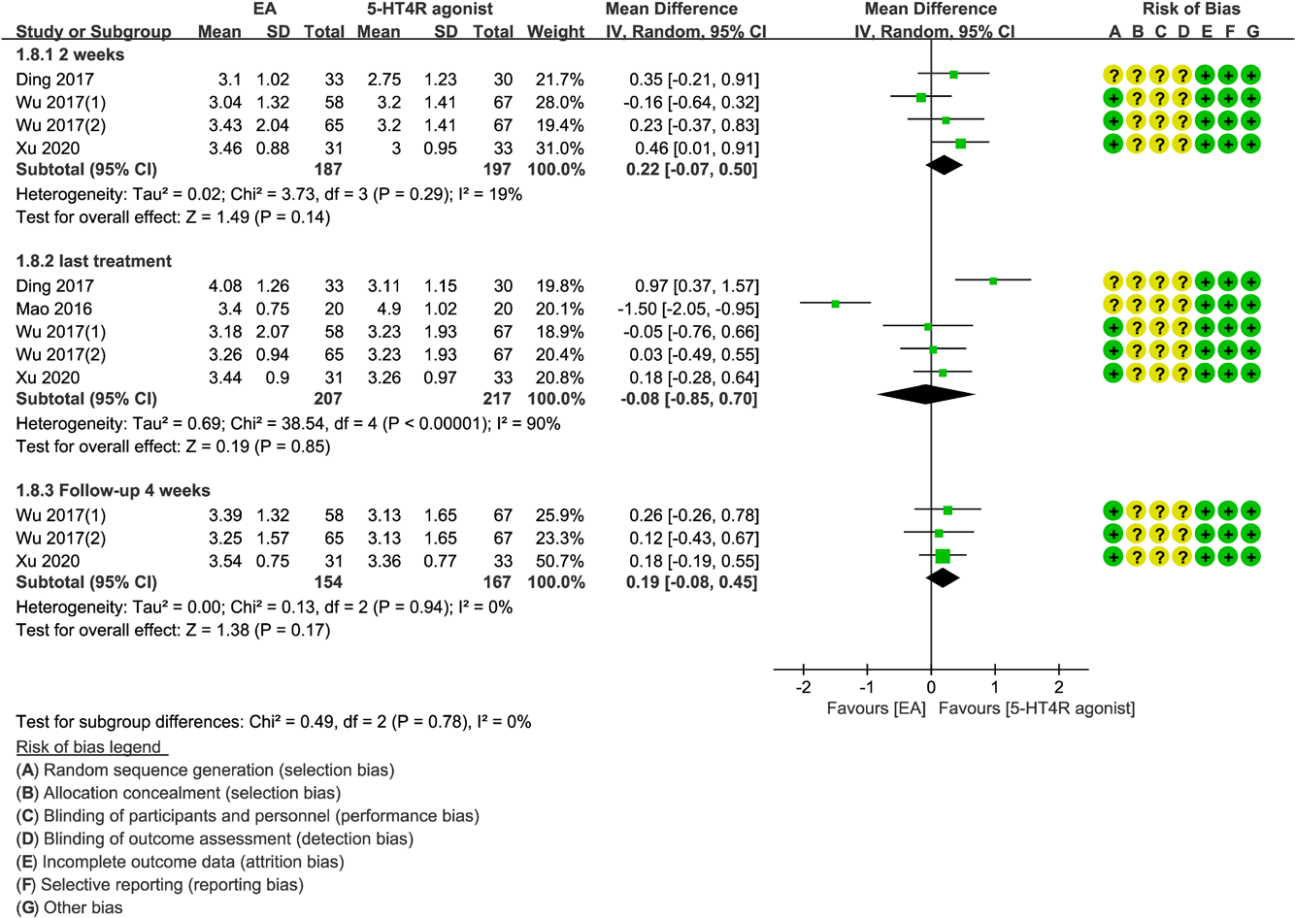
Forest plot of EA versus 5-HT4 receptor agonist stool consistency (2 weeks, last treatment and follow-up 4 weeks).

We determined stool consistency at Bristol stool form scale. As indicated by the results, there were superior effects of stool consistency in the EA group in comparison to the control in the fourth week of follow-up and the second week of treatment. In the last treatment, the stool consistency of the control was better the EA group, whereas the heterogeneity was very high (*I*^2^ =90). However, not any statistical difference was found between the 3 groups (MD = 0.22; 95% CI = −0.07 to 0.50, *P* = .14; MD = −0.08; 95% CI = −0.85 to 0.70, *P* = .85; MD = 0.19; 95% CI = −0.08 to 0.45, *P* = .17, respectively) (Fig. 5).

### 3.7. Patient assessment of constipation quality of life questionnaire

**Figure 6.**
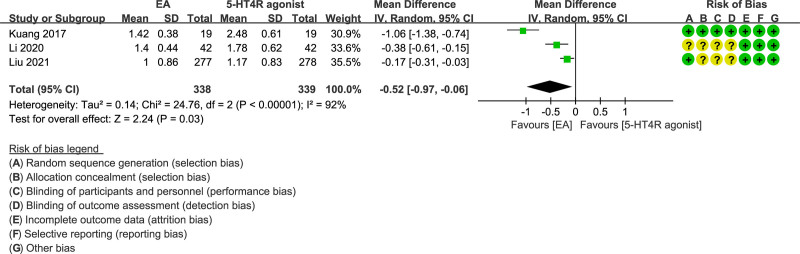
Forest plot of EA versus 5-HT4 receptor agonist PAC-QOL.

Three studies^[26,33,36]^ with 677 subjects in total investigated the PAC-QOL score. The impairment of quality of life in relation to health in patients with constipation was similar to that of ulcerative colitis.^[38]^ As revealed by the results, patients who have received EA had a lower PAC-QOL score than those treated with 5-HT4 receptor agonist (MD = −0.52; 95% CI = −0.97 to −0.06, *P* = .03) (Fig. 6).

### 3.8. self-rating anxiety scale and self-rating depression scale

**Figure 7.**
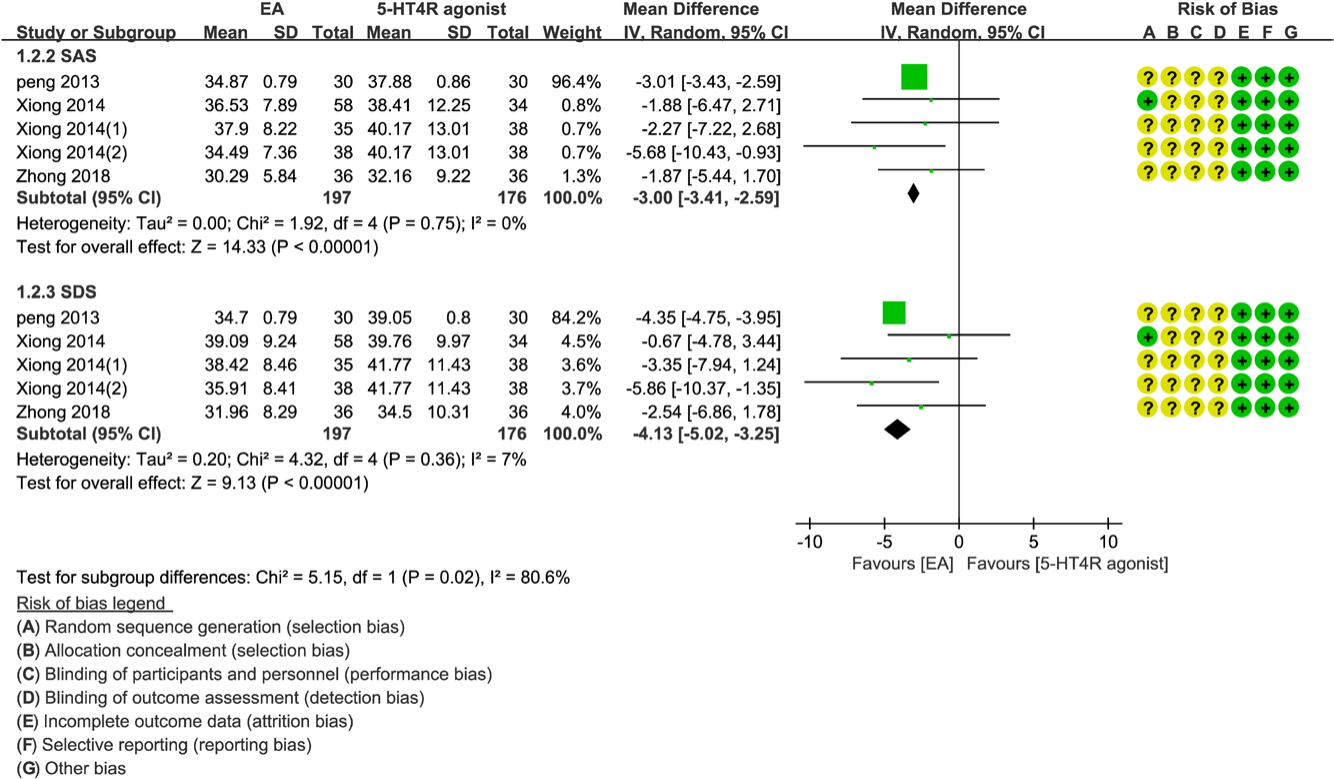
Forest plot of EA versus 5-HT4 receptor agonist SAS and SDS.

We employed SAS and SDS for an evaluation of the degree of anxiety and depression. The lower the score, the better the mental state would be. As expected, the results indicated that EA group had a lower SAS and SDS score than the control (MD = −3.00; 95% CI = −3.41 to −2.59, *P* < .00001; MD = −4.13; 95% CI = −5.02 to −3.25, *P* < .00001, respectively) (Fig. 7).

### 3.9. Adverse events

Four RCTs^[26,30,34,37]^ reported adverse events specifically. Only 3 of the 12 studies (with 510 patients involved) reported that the EA group had an adverse event, and 55 cases were overall identified (55/510, 10.78%). The most frequently reported adverse events in the EA group, including pain, local hematoma and dizziness. However, 4 studies (with 403 patients involved) reported 136 cases of adverse events in the control (136/403, 33.75%). Furthermore, a study reported that no serious adverse events occurred.

### 3.10. Publication bias

Since there are fewer than 10 studies that drew a comparison of the outcomes, publication bias was not assessed.

### 3.11. Evidence quality

GRADEpro (https://gdt.gradepro.org/app/) was exploited to assess the evidence quality in meta-analysis. All outcomes’ evidence quality was low due to bias risk, small samples and high heterogeneity (Table 3).

**Table 3:** Summary of findings, based on GRADEpro.

Certainty assessment					
Outcomes	No. of participants (studies)	Certainty of the evidence (GRADE)	Relative effect (95% CI)	Anticipated absolute effects	
				Risk with 5-HT4 receptor agonist	Risk difference with EA
SAS	373 (5 RCTs)	⊕⊕⚪⚪ **LOW**†,‡	-	-	MD **3 lower** (3.41 lower to 2.59 lower)
SDS	373 (5 RCTs)	⊕⊕⚪⚪** LOW**†,‡	-	-	MD **4.13 lower** (5.02 lower to 3.25 lower)
CSBMs (follow-up 4 wk)	595 (2 RCTs)	⊕⊕⚪⚪** LOW**†,‡	-	-	MD **0.05 higher** (0.14 lower to 0.25 higher)
CSBMs (follow-up 8 wk)	595 (2 RCTs)	⊕⊕⚪⚪** LOW**†,‡	-	-	MD **0.06 highe**r (0.26 lower to 0.39 higher)
CSBMs (last treatment)	595 (2 RCTs)	⊕⊕⚪⚪** LOW**†,‡	-	-	MD **0.06 higher** (0.12 lower to 0.25 higher)
PAC-QOL	677 (3 RCTs)	⊕⊕⚪⚪** LOW**‡,$	-	-	MD** 0.52 lower** (0.97 lower to 0.06 lower)
LOW† (2 wk)	1103 (7 RCTs)	⊕⊕⚪⚪** LOW**†	-	-	MD **0.09 higher** (0.43 lower to 0.60 higher)
LOW† (last treatment)	588 (7 RCTs)	⊕⊕⚪⚪** LOW**†,‡	-	-	MD **0.41 higher** (0.10 lower to 0.93 higher)
LOW† (follow-up 4 wk)	485 (5 RCTs)	⊕⊕⚪⚪ LOW*	-	-	MD **0.19 higher** (0.29 lower to 0.67 higher)
Stool consistency (2wk)	384 (4 RCTs)	⊕⊕⚪⚪** LOW**†,‡	-	-	MD **0.22 higher** (0.07 lower to 0.50 higher)
Stool consistency (last treatment)	424 (5 RCTs)	⊕⊕⚪⚪** LOW**†	-	-	MD **0.08 lower** (0.85 lower to 0.70 higher)
Stool consistency (follow-up 4 wk)	321 (3 RCTs)	⊕⊕⚪⚪** LOW**†,‡	-	-	MD **0.19 higher** (0.08 lower to 0.45 higher)

Annotation: The risk in the intervention group (and its 95% confidence interval) is based on the assumed risk in the comparison group and the relative effect of the intervention (and its 95% CI).CI = confidence interval, MD = mean difference. GRADE Working Group grades of evidence. High certainty: we are very confident that the true effect lies close to that of the estimate of the effect. Moderate certainty: we are moderately confident in the effect estimate: the true effect is likely to be close to the estimate of the effect, but there is a possibility that it is substantially different; Low certainty: our confidence in the effect estimate is limited: the true effect may be substantially different from the estimate of the effect; Very low certainty: we have very little confidence in the effect estimate: the true effect is likely to be substantially different from the estimate of effect.

† Due to risk of bias.

‡ Due to Imprecisior.

$ Due to high heterogeneity.

## 4. Discussion

Generally, there are many drugs to treat constipation (such as 5-HT4 receptor agonists), but most patients were not satisfied with the current relief therapy.^[39]^ In China, the complementary application of electroacupuncture is extremely common in patients with constipation. However, whether they are actually clinically effective, and the mechanism of action is unknown. Accordingly, we made a meta-analysis concerned with relevant RCTs to carry out a reliable quantitative assessment of the existing evidence on the efficacy of EA versus 5-HT4 receptor agonists for treating FC.

In this paper, 12 RCTs involving 1473 patients with FC were analyzed. We collected data on various parameters, including weekly CSBMs, weekly SBMs, stool consistency, PAC-QOL, SAS, SDS, and adverse events (AEs), to evaluate improvements in multiple aspects of FC. The main results indicated that both EA and 5-HT4 receptor agonists can improve weekly CSBMs, weekly SBMs, and stool consistency, although the differences were not statistically significant. However, the EA group showed greater improvement in PAC-QOL, SAS, and SDS compared to the control group. Due to the small number of included studies, the interpretation of these results should be approached with caution. Additionally, the EA group had a significantly lower incidence of adverse events compared to the control group. The evidence indicates that electroacupuncture (EA) has a therapeutic effect comparable to that of 5-HT4 receptor agonists in treating FC. However, EA is more effective than 5-HT4 receptor agonists in enhancing the quality of life and mental state of FC patients, while also exhibiting fewer side effects. This information serves as a valuable reference for clinicians.

Currently, acupuncture is widely used to treat various gastrointestinal disorders. The potential mechanisms of acupuncture include neural regulation, modulation of gastrointestinal motility and visceral hypersensitivity, anti-inflammatory effects, restoration of gut microbiota, and improvement of the intestinal barrier.^[40]^ Intestinal motility is primarily regulated by excitatory and inhibitory neurons.^[41]^ Experimental studies have demonstrated that electroacupuncture (EA) stimulates the release of acetylcholine in the colon via the central afferent-cholinergic pathway, effectively promoting gastrointestinal peristalsis in rats.^[42]^ Butyrate, an important metabolite of human intestinal flora, can alleviate FC by promoting intestinal contraction and peristalsis, exhibiting anti-inflammatory properties, and enhancing the intestinal barrier.^[43]^ Further research has shown that EA treatment increases butyrate, an important metabolite produced by intestinal microbiota, and restores the composition and abundance of intestinal flora in mice with FC.^[44]^ Clinical studies indicate that effective needling similarly enhances butyrate levels in the human intestinal tract, restoring the composition of the intestinal microbiota and leading to improvements in FC.^[45]^ Our study found that electroacupuncture improved weekly CSBMs, fecal consistency, mood disorders, and quality of life in patients with FC, likely related to the aforementioned mechanisms.

It is noteworthy that EA was comparable to 5-HT4 receptor agonists in improving constipation-related symptoms. In the included studies, the control mainly consisted of Mosapride cirate and prucalopride. Prucalopride was a highly selective stimulant for constipation,^[46]^ stimulating peristalsis of intestinal smooth muscles through the stimulation of 5-HT receptors on intestinal neurons and the release of acetylcholine. Moreover, EA may regulate the 5-HT system in animal experiments.^[47]^ In addition, EA significantly improved fatigue, anxiety, and depression in RCT of breast cancer patients suffering from drug-related joint pain.^[48]^ EA also relieved drug-induced depression-like behavior in rats.^[49]^ Our results indicated that EA could better improve patients’ quality of life and reduce anxiety and depression than 5-HT4 receptor agonists.

## 5. Limitations

While the results suggest that EA offers advantages over 5-HT4 receptor agonists in constipation-related indicators, this study has several limitations: Many studies only briefly mention “random” without detailing the specific method of random sequence generation. There is limited research on blinding of outcome assessment, blinding of participants and personnel, and allocation concealment, which may compromise the reliability of high-quality RCTs. The study subjects are exclusively Chinese. Although EA is widely used clinically in China, only 4 papers were published in English. Given that constipation affects all races, biases related to reporting and language are likely present in the results. Some trials included in this meta-analysis had small sample sizes, potentially leading to an overestimation of the therapeutic effect. Publication bias was not assessed due to the relatively small number of trials. Acupoints are specific, and variations in acupoint selection across trials may introduce selection biases.

## 6. Conclusion

Overall, based on the findings, electroacupuncture (EA) as an alternative therapy appears to demonstrate comparable efficacy to 5-HT4 receptor agonists and may contribute to improvements in PAC-QOL, SAS, and SDS among patients with FC. In this study, we employed the GRADE method to assess the quality of included RCTs. However, due to the scarcity of high-quality trials, the primary outcomes were deemed to have a low certainty level. Therefore, we strongly advocate for additional high-quality RCTs to delve deeper into acupoint specificity and assess long-term maintenance effects. Further research in this area can provide valuable insights for practitioners aiming to integrate EA with conventional and modern medical treatments.

## Author contributions

**Conceptualization:** Jiacheng Li, Aimei Wang.

**Data curation:** Shanchun Xu, Jiacheng Li.

**Funding acquisition:** Shanchun Xu, Jiacheng Li.

**Methodology:** Aimei Wang.

**Writing – original draft:** Shanchun Xu, Jiacheng Li.
